# Retraction Note: Visnagin alleviates rheumatoid arthritis via its potential inhibitory impact on malate dehydrogenase enzyme: in silico, in vitro, and in vivo studies

**DOI:** 10.1186/s12263-025-00787-4

**Published:** 2025-11-13

**Authors:** Abeer A. Khamis, Amira H. Sharshar, Tarek M. Mohamed, Elsayed A. Abdelrasoul, Maha M. Salem

**Affiliations:** 1 Biochemistry Division, Chemistry Department, Faculty of Science, Tanta University, Tanta, Egypt; 2 Head Researcher of Special Food and Nutrition Department, Food Technology Research Institute, Agricultural Research Center, Giza, Egypt

**Retraction Note: Genes & Nutrition (2024) 19:20**

10.1186/s12263-024-00756-3

The Editor-in-Chief has retracted this article. After publication, concerns were raised regarding Fig. 5. Specifically, A and B appear to overlap, and D, E and G appear to overlap. The Authors submitted the wide view images upon request, and these did not alleviate concerns. The Editor-in-Chief has lost confidence in the data and conclusions of this article.

All authors disagree with this retraction.

